# Differential Induction and Resuscitation of the Viable but Non-Culturable (VBNC) State in *Klebsiella pneumoniae* by Sodium Hypochlorite and Glutaraldehyde: Insights from Energy Metabolism and Antioxidant Systems

**DOI:** 10.3390/microorganisms14040905

**Published:** 2026-04-17

**Authors:** Chengwei Li, Honglin Ren, Yuanyuan Zhang, Ruoran Shi, Bo Zhang, Shaohui Hu, Jiaqi Hou, Ziqi Xing, Yuyang Ding, Fang Yang, Yansong Li, Shiying Lu, Qiang Lu, Zengshan Liu, Xiaoxu Wang, Pan Hu

**Affiliations:** 1 State Key Laboratory for Diagnosis and Treatment of Severe Zoonotic Infectious Diseases, Key Laboratory for Zoonosis Research of the Ministry of Education, Institute of Zoonosis, College of Veterinary Medicine, Jilin University, Changchun 130062, China; 2 Institute of Special Animal and Plant Sciences, Chinese Academy of Agricultural Sciences, Changchun 130112, China

**Keywords:** *Klebsiella pneumoniae*, viable but non-culturable (VBNC), sodium hypochlorite, glutaraldehyde, resuscitation

## Abstract

This study systematically compared the induction and resuscitation characteristics of the viable but non-culturable (VBNC) state in *Klebsiella pneumoniae *FY170-1 following sublethal exposure to sodium hypochlorite (NaClO) or glutaraldehyde (GA). Treatment with 30 mg/L NaClO or 60 mg/L GA for 60 min reduced culturability to below the detection limit (<1 CFU/mL). However, CTC staining showed that 50.80% and 63.44% of cells, respectively, retained respiratory activity, while SYTO 9/PI staining indicated that membrane integrity was largely preserved, consistent with induction of the VBNC state. Scanning electron microscopy revealed distinct morphological alterations in the two groups. NaClO-induced VBNC cells showed surface depressions and wrinkling, consistent with oxidative damage, whereas GA-induced cells exhibited filamentous and net-like surface structures, consistent with aldehyde-mediated cross-linking. Among the tested additives, sodium succinate showed the strongest resuscitation-promoting effect under the experimental conditions, with OD600 increasing after approximately 2 h of incubation. Post-resuscitation analysis further revealed marked differences between the two VBNC states. In resuscitated NaClO-induced VBNC cells, ATP partially recovered, but reactive oxygen species remained elevated and catalase activity showed little recovery. In contrast, resuscitated GA-induced VBNC cells exhibited lower ATP recovery but more rapid normalization of ROS and better recovery of oxidative stress-related parameters. Total protein analysis and SDS-PAGE further supported distinct patterns of protein-level alteration between the two treatments. Overall, these findings suggest that NaClO and GA induce phenotypically distinct VBNC states in *K. pneumoniae*, with different recovery behaviors and stress response profiles. Sodium succinate was identified as the most effective recovery-promoting additive under the tested conditions. These results highlight the risk of underestimating bacterial survival when culturability is used as the sole indicator of disinfection efficacy and support the need for more comprehensive viability assessment.

## 1. Introduction

Pathogenic bacteria encountered during food processing, cold-chain transport, and environmental disinfection are routinely exposed to multiple stresses, including low temperature, nutrient limitation, osmotic pressure fluctuations, and disinfectant exposure. To survive such adverse conditions, many bacteria enter a specialized survival state known as the viable but non-culturable (VBNC) state [1]. VBNC cells fail to form colonies on standard culture media and are therefore undetectable by conventional plate-counting methods, yet they typically retain intact cell membranes along with residual metabolic activity and the potential to express pathogenicity-related factors [2]. When external conditions become favorable, VBNC cells can resuscitate, regaining culturability and proliferative capacity and thereby creating cryptic contamination sources and potential health risks within food systems and processing environments [3]. The mechanisms governing VBNC formation and resuscitation have therefore become a central concern in food microbial safety, especially in settings that rely on culture-based assays for disinfection efficacy evaluation and hygienic monitoring, where VBNC populations may produce false-negative results [4].

*Klebsiella pneumoniae* is a major Gram-negative opportunistic pathogen that is strongly associated with human respiratory tract infections, urinary tract infections, and bacteremia, and is also capable of long-term persistence in animal husbandry environments, wastewater, and food-related settings [5]. The organism displays pronounced environmental adaptability and persistence, conferred by its capsule, diverse adhesion structures, and robust biofilm-forming capacity, which enable it to withstand cleaning and disinfection, desiccation, and nutrient limitation more effectively in complex environments [6,7]. Of greater concern is that *K. pneumoniae* can transition into the VBNC state under suboptimal conditions and resuscitate when conditions are restored, thereby presenting significant challenges to risk control and disinfection efficacy assessment in food production [8]. Nevertheless, compared with well-characterized model organisms such as *Escherichia coli* and *Vibrio species*, studies on disinfectant-induced VBNC formation and resuscitation in *K. pneumoniae* remain relatively scarce, particularly those providing systematic multi-indicator datasets.

In food and environmental hygiene control, chemical disinfection continues to be one of the most widely used and essential interventions. Sodium hypochlorite (NaClO), a chlorine-based disinfectant applied extensively, acts primarily through strong oxidation to damage microbial proteins and enzyme systems, whereas glutaraldehyde (GA), an aldehyde-class high-level disinfectant, cross-links protein amino groups and thereby inactivates microbial structure and function [9,10]. Although both agents exhibit potent bactericidal activity, practical applications are often subject to sublethal exposure caused by fluctuating disinfectant concentrations, organic load, insufficient contact time, inaccessible micro-niches, or non-standard procedures, thereby imposing sustained “non-lethal pressure” on bacterial populations [11,12,13]. Existing research has identified sublethal stress as a key trigger for inducing the VBNC state; consequently, “incomplete disinfection” not only signifies inadequate inactivation but may actively promote pathogen entry into VBNC followed by resuscitation under permissive conditions, generating risks of persistent contamination [14]. In the context of food processing and environmental disinfection, this risk requires rigorous quantification and mechanistic clarification.

Against this background, the present study utilized the environmentally isolated *Klebsiella pneumoniae* strain FY170-1 to construct experimental models of sodium hypochlorite- and glutaraldehyde-induced viable but non-culturable states and systematically assessed the ability of the two disinfectants to induce this state under sublethal conditions together with the associated resuscitation characteristics. Specifically, conditions resulting in complete loss of culturability were first established by plate counting, and the validity of the state was confirmed at the levels of membrane integrity and respiratory metabolism using fluorescence microscopy and flow cytometry. Scanning electron microscopy was subsequently employed to compare differences in cellular ultrastructural remodeling induced by the two disinfectants, thereby revealing distinct damage pathways. On this basis, resuscitation kinetic curves were used to screen and identify sodium succinate as the most effective resuscitation promoter. Quantitative analyses of reactive oxygen species and ATP then revealed changes in oxidative stress and energy metabolism during both the VBNC state and resuscitation. Catalase and superoxide dismutase activities were measured to evaluate impairment and reconstruction of the cellular antioxidant defense system. Finally, BCA protein quantification and SDS-PAGE described overall protein-level alterations, thereby establishing a multidimensional evidence chain spanning culturability, cellular structure, metabolic activity, antioxidant function, and protein phenotype. Through integrated analysis, this study aims to elucidate the differential damage pathways and resuscitation mechanisms elicited by sodium hypochlorite versus glutaraldehyde in inducing the viable but non-culturable state, providing experimental evidence and theoretical reference for disinfection efficacy assessment and control of potential resuscitation risks in food-related environments.

## 2. Materials and Methods

### 2.1. Bacterial Strains and Culture Conditions

Strain *Klebsiella pneumoniae *FY170-1, an environmental isolate from our laboratory, was purified through standard isolation and identification procedures, verified by whole-genome sequencing, and stored as a glycerol stock at −80 °C. After thawing on ice, the stock was streaked onto LB agar plates and incubated at 37 °C for approximately 20 h. A single colony was inoculated into 5 mL of LB broth and grown overnight at 37 °C with shaking at 180 rpm. The overnight culture was diluted 1:100 into 100 mL of LB broth and grown at 37 °C with shaking at 180 rpm to the logarithmic phase for subsequent experiments.

### 2.2. Determination of Minimum Inhibitory Concentration (MIC) and Minimum Bactericidal Concentration (MBC)

The MIC of NaClO against *Klebsiella pneumoniae *FY170-1 was determined using the 96-well microtiter broth dilution method [15]. Disinfectant solutions prepared from NaClO (5% available chlorine) and 2% glutaraldehyde were serially twofold diluted in 96-well plates and supplemented with LB to yield final concentrations ranging from 512 to 0.5 mg/L. OD600 was measured at 0 h and 24 h; the lowest disinfectant concentration producing an OD difference <0.05 was defined as the MIC. Culture systems containing the MIC and higher disinfectant concentrations were incubated at 37 °C for 24 h and then plated onto LB agar for CFU enumeration; the lowest concentration yielding no visible colonies was defined as the MBC. Each experiment was performed in triplicate.

### 2.3. Induction of the Viable but Non-Culturable (VBNC) State in Klebsiella pneumoniae by Sodium Hypochlorite and Glutaraldehyde

*Klebsiella pneumoniae* was cultured for 4 h to reach the logarithmic growth phase. Two milliliters of the culture were harvested by centrifugation at 12,000× *g* for 10 min, washed with sterile PBS to remove residual medium, and adjusted to the required concentration. A 10 mL induction system was prepared by combining sodium hypochlorite solution (5% available chlorine) with sterile PBS, to which the washed cells were added to achieve a final available chlorine concentration of 30 mg/L and a cell density of 1 × 10^7^ CFU/mL. A second 10 mL induction system was prepared using freshly prepared 2% glutaraldehyde solution and sterile PBS, with washed cells added to reach a final glutaraldehyde concentration of 60 mg/L and 1 × 10^7^ CFU/mL. The induction mixtures were vortexed and incubated in the dark at 25 °C with shaking at 120 rpm. Samples (1 mL) were withdrawn every 10 min; for the NaClO group, sodium thiosulfate was added immediately to quench the chlorination reaction. Each sample was centrifuged at 12,000× *g* for 2 min, washed twice with physiological saline, and resuspended in 1 mL of physiological saline. One hundred microliters were plated onto LB agar. After incubation at 37 °C for 24 h, culturable counts (CFU) were recorded. When no visible colonies appeared (i.e., when the concentration of culturable cells was below 10 CFU/mL), the bacterial suspension used for plating was concentrated 10-fold and re-plated. When no visible colonies were observed after re-plating (i.e., when the concentration of culturable cells was below 1 CFU/mL), all cells were considered to have entered a non-culturable state [16]. This experiment was repeated six times. After the conditions were confirmed to be stably reproducible, they were used for the large-scale preparation of VBNC samples in subsequent experiments. For each subsequent batch of prepared samples, the spread plate method was likewise used to determine whether any residual culturable bacteria remained.

### 2.4. Confocal Laser Scanning Microscopy

To assess the survival status and physiological characteristics of *K. pneumoniae* cells that had entered the VBNC state following sodium hypochlorite or glutaraldehyde treatment, CTC (5-cyano-2,3-ditolyl tetrazolium chloride) staining was performed for respiratory metabolism and SYTO 9/propidium iodide (PI) staining for membrane integrity, with imaging conducted by confocal laser scanning microscopy.

Cell activity was detected using CTC (MCE, Shanghai, China). CTC is reduced by the respiratory electron transport chain to form insoluble red fluorescent formazan (CTC-formazan), thereby labeling metabolically active cells. Two hundred microliters of bacterial suspension were mixed with 20 μL of prepared CTC working solution (final concentration 5 mmol/L), gently mixed, and incubated in the dark at 37 °C for 60 min. After incubation, cells were pelleted by centrifugation at 12,000× *g* for 5 min, washed twice with sterile PBS to remove unbound dye, and resuspended in 200 μL of PBS. Ten microliters were placed on a clean glass slide, covered with a coverslip, and imaged immediately. CTC-formazan was excited at 561 nm with emission collected at 570–620 nm; red fluorescence signals indicate metabolically active cells.

Membrane integrity was assessed using the LIVE/DEAD BacLight Bacterial Viability Kit (Thermo Fisher Scientific, Waltham, MA, USA). SYTO 9 and PI were mixed to prepare the working solution according to the manufacturer’s instructions. Briefly, 3 μL of the dye mixture was added to 1 mL of cell suspension, gently mixed, and incubated in the dark at 25 °C for 15 min. After incubation, 10 μL of the stained suspension was placed on a clean glass slide for observation. SYTO 9 was excited at 488 nm, and its emission was collected at 500–550 nm; PI was excited at 561 nm, and its emission was collected at 570–620 nm. SYTO 9 penetrates all cells and emits green fluorescence, whereas PI enters only cells with damaged membranes and emits red fluorescence. Therefore, under overlapping fields of view, green signals indicate viable cells, while red signals indicate dead or membrane-damaged cells.

In this study, the bacterial concentration was adjusted prior to staining, and these two assays were used as qualitative or semi-quantitative imaging methods to compare the relative changes in metabolic activity and membrane integrity among treatments, rather than for absolute quantitative determination.

### 2.5. Enumeration of VBNC Cells

Quantitative analysis of cells in different physiological states was performed by CTC staining combined with flow cytometry using a BD FACSVerse flow cytometer (BD Biosciences, Franklin Lakes, NJ, USA). The CTC staining procedure was the same as that described in Section 2.4. Untreated and unstained bacterial cells were used as controls for instrument parameter adjustment. After staining, the cells were resuspended in 200 μL PBS and analyzed by flow cytometry. CTC-formazan was excited at 561 nm, and the red fluorescence signal was collected at 570–620 nm. Red fluorescence was considered indicative of metabolically active cells.

### 2.6. Morphological Analysis of VBNC Cells

Following induction of *K. pneumoniae* into the VBNC state by sodium hypochlorite or glutaraldehyde, bacterial suspensions were collected and centrifuged at 12,000× *g* for 10 min at 4 °C. The supernatant was discarded, and cells were fixed with 2.5% glutaraldehyde at 4 °C for 24 h. Fixed samples were washed twice with PBS, dehydrated through a standard ethanol gradient, and dried. Dried specimens were sputter-coated with gold and examined under a scanning electron microscope (SEM, Hitachi S-3400N, Tokyo, Japan). Morphological differences between VBNC cells under each induction condition and untreated control cells were compared.

### 2.7. Resuscitation of VBNC-State Klebsiella pneumoniae

Resuscitation capacity of VBNC *K. pneumoniae* was evaluated with reference to previously reported protocols with appropriate modifications [16]. Four different resuscitation regimens were tested by supplementing LB broth with distinct additives to compare their ability to promote recovery of VBNC cells (Table 1). For Autoinducer-2 (AI-2) supplementation, cell-free supernatant from *Escherichia coli* MG1655 cultures was used. MG1655 was cultured for 5 h, followed by centrifugation to collect the supernatant, which was subsequently filtered through a 0.22 μm membrane to remove bacterial cells. For the homologous supernatant, *Klebsiella pneumoniae *FY170-1 was cultured for 4 h, after which the culture was centrifuged and the supernatant collected. The supernatant was then filtered through a 0.22 μm membrane to remove bacterial cells. Cells induced into the VBNC state by sodium hypochlorite or glutaraldehyde, together with untreated controls, were harvested by centrifugation and washed with sterile physiological saline. To minimize interference from residual culturable cells, all resuscitation samples were serially diluted twice prior to use. Treated suspensions were inoculated into 10 mL LB broth containing the respective additives; LB medium without any additives and uninoculated negative controls were included. Cultures were incubated with shaking at 37 °C and 180 rpm for 25 h, and OD600 was measured using a microplate reader upon visible growth. To monitor resuscitation kinetics, a parallel micro-culture system was established in 96-well plates in a temperature-controlled multi-mode automatic microplate reader, with OD600 recorded every 30 min to generate growth curves. Resuscitation was confirmed when no growth occurred in the additive-free LB control or uninoculated negative control groups and when results were consistent between the 10 mL and 96-well systems. Each experiment included three parallel replicates; all treatment groups were independently repeated three times to ensure reliability and reproducibility.

### 2.8. Measurement of Reactive Oxygen Species (ROS) Levels

Intracellular reactive oxygen species (ROS) levels were quantified using the ROS Assay Kit from Beyotime Biotechnology Co., Ltd. (Shanghai, China). Bacteria induced into the VBNC state by sodium hypochlorite or glutaraldehyde, along with sodium hypochlorite-treated VBNC cells resuscitated with sodium succinate for 18 h (Resuscitated CL-pretreated Cells, RCL) and glutaraldehyde-treated VBNC cells resuscitated for 18 h (Resuscitated GA-pretreated Cells, RGA), were collected and subjected to Percoll density-gradient centrifugation to reduce dead-cell contamination (i.e., reduce the proportion of dead cells). Uninduced *Klebsiella pneumoniae* cells served as the viable control (Control), while heat-killed (boiled) cells served as the dead control (Dead). Bacterial samples from each group were washed with PBS, resuspended, and normalized to 1 × 10^7^ CFU/mL. Two hundred microliters of suspension were mixed with working solution containing the fluorescent probe DCFH-DA, transferred to a black 96-well plate, and incubated at 37 °C in the dark for 20 min. Fluorescence intensity was subsequently measured on a fluorescence microplate reader (excitation 488 nm, emission 525 nm). ROS levels were expressed as relative fluorescence intensity (RFI). All experiments were conducted independently in triplicate.

### 2.9. Measurement of ATP Levels

Intracellular ATP levels were determined using the BacTiter-Lumi™ Luminescent Microbial Cell Viability Assay Kit (Beyotime Biotechnology, Shanghai, China). The assay is based on a luciferase-catalyzed ATP-dependent bioluminescence reaction, enabling quantitative analysis of ATP through chemiluminescence intensity. Cells induced into the VBNC state by sodium hypochlorite or glutaraldehyde, together with cells resuscitated for 18 h, were collected and subjected to Percoll density-gradient centrifugation to reduce dead-cell contamination. Uninduced *K. pneumoniae* cells served as the viable control, and heat-killed (boiled) cells served as the dead control. Following the manufacturer’s protocol, bacterial suspensions were mixed with an equal volume of BacTiter-Lumi™ reagent and incubated at room temperature in the dark for 30 min. Luminescence was measured using a multi-mode microplate reader (TECAN Spark, Tecan Group Ltd., Männedorf, Switzerland). ATP levels were reported as relative luminescence units (RLU). All experiments were performed independently in triplicate.

### 2.10. Measurement of Antioxidant Enzyme Activities

Superoxide dismutase (SOD) and catalase (CAT) activities were assayed using the Solarbio SOD Assay Kit and Solarbio CAT Assay Kit (Solarbio, Beijing, China), respectively. Experimental groups comprised positive control, negative control, CL, GA, RCL, and RGA groups, each with three biological replicates. The CL group consisted of cells induced into the VBNC state by sodium hypochlorite, the GA group consisted of cells induced by glutaraldehyde, and the RCL and RGA groups comprised the corresponding CL and GA cells after 18 h resuscitation with sodium succinate; group designations followed those used in prior sections. For sample preparation, cells were pelleted by centrifugation at 4 °C and 12,000 r/min for 10 min, resuspended in PBS, and standardized to approximately 1 × 10^7^ CFU/mL. To release intracellular proteins for enzymatic assays, cells were lysed by sonication on ice, followed by centrifugation at 12,000 r/min for 10 min at 4 °C; the resulting supernatant served as the enzyme assay sample. SOD activity was measured according to the Solarbio kit instructions: after mixing supernatant with the reaction system and incubating at 37 °C for 30 min, absorbance was read at 450 nm on a microplate reader, and activity was calculated using the kit-provided formula and expressed in U/mL. CAT activity was determined per the Solarbio kit protocol: after substrate reaction, absorbance was read at 240 nm, and activity was calculated and expressed in U/mL. All experiments were performed independently in triplicate.

### 2.11. Protein Content and Expression Analysis in VBNC-State Klebsiella pneumoniae

Protein extraction from VBNC-state *Klebsiella pneumoniae* was performed using the SDC ProPrep Kit (Majorbio Bio-Pharm Technology Co., Ltd., Shanghai, China) to determine total protein content and examine expression profiles. Samples were placed in 2 mL grinding tubes with pre-chilled lysis buffer (Lysis I) and one 6 mm steel bead, wet-ground for 180 s per cycle (three cycles), and then vortexed at 95 °C and 1500 rpm for 5 min. After centrifugation at 4 °C and 14,000× *g* for 10 min, the supernatant was collected for downstream analysis. Protein concentration was quantified with the Pierce BCA Protein Assay Kit (Thermo Fisher Scientific, Waltham, MA, USA) using a BSA standard curve (0, 0.125, 0.25, 0.5, 0.75, 1, 1.5, 2 μg/μL); absorbance was read at 562 nm on a microplate reader (SPECTRA MAX, Molecular Devices, San Jose, CA, USA), and concentrations were calculated from the standard curve. SDS-PAGE was subsequently performed: samples 4–6 were loaded at 10 μL, while remaining samples were loaded at 10 μg protein per lane. Samples were mixed 4:1 with 5× SDS loading buffer, denatured at 95 °C for 5 min, and electrophoresed on 5% stacking and 12% separating gels (80 V through the stacking gel, followed by constant 120 V through the separating gel until the bromophenol blue dye front reached the gel bottom). Gels were stained with Coomassie Brilliant Blue R-250 (Solarbio Science & Technology Co., Ltd., Beijing, China) for 1 h and destained in methanol:glacial acetic acid:distilled water (4:1:5) until the background was clear. Differences in protein band patterns among treatment groups were recorded. All experiments were performed independently in triplicate.

### 2.12. Statistical Analysis

All quantitative data are expressed as mean ± standard deviation (mean ± SD). Statistical analyses were conducted using SPSS 22.0 (IBM Corp., Armonk, NY, USA). Inter-group differences were assessed by one-way ANOVA followed by Tukey’s multiple comparisons test with correction for multiple comparisons. Differences were considered statistically significant at *p* < 0.05. Graphs were generated using Origin 2019b (OriginLab Corp., Northampton, MA, USA).

## 3. Results

### 3.1. MIC and MBC of NaClO and GA Against Klebsiella pneumoniae FY170-1

**Figure 1:**
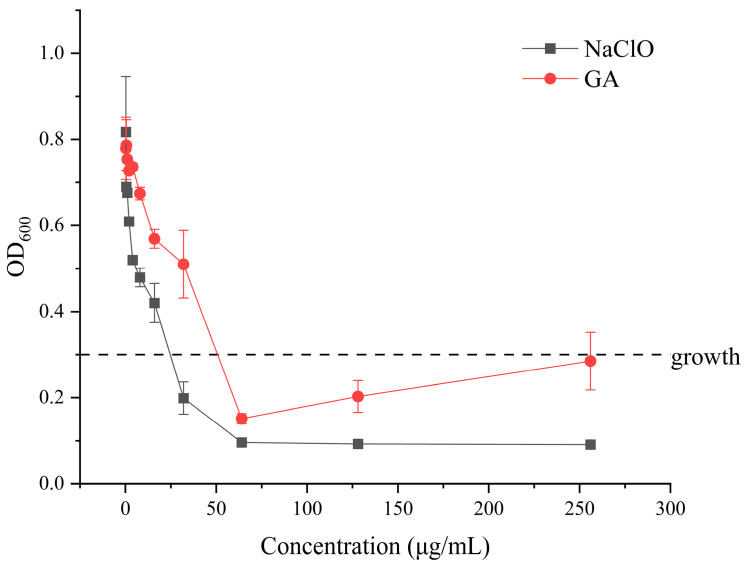
OD600 values of *Klebsiella pneumoniae* after 24 h culture under different concentrations of sodium hypochlorite/glutaraldehyde.

Using the 96-well microtiter broth dilution method, the minimum inhibitory concentration (MIC) of sodium hypochlorite against this strain was determined to be 32 mg/L, whereas the MIC of glutaraldehyde was 64 mg/L (Figure 1). The minimum bactericidal concentration (MBC) was further determined using disinfectant concentrations at and above the MIC (32, 64, 128, 256, and 512 mg/L). After plating and incubation at 37 °C for 24 h, the MBC of sodium hypochlorite against this strain was found to be 64 mg/L, while the MBC of glutaraldehyde was 128 mg/L. For both disinfectants, the MBC exceeded the MIC, with the MBC being approximately twofold higher than the MIC, indicating that reaching the MIC does not necessarily result in complete cell inactivation. Based on these findings, the MIC and MBC values were used as reference doses for establishing the VBNC induction model in this study. Sublethal concentrations close to the MIC and MBC were selected to promote the gradual transition of cells from a culturable state to a viable but non-culturable (VBNC) state, while minimizing their direct progression to complete cell death. In the experiments, excessively high glutaraldehyde concentrations were found to darken the LB medium and elevate absorbance, causing the MIC curve for glutaraldehyde to rise at high concentrations. At these points, the medium remained clear and transparent, and plate counts showed no bacterial growth, confirming that the MIC rebound resulted from cross-linking reactions (e.g., Schiff base formation) between glutaraldehyde and proteins or amino acids in LB rather than bacterial proliferation [17].

### 3.2. Conditions and Validation of VBNC Induction in Klebsiella pneumoniae by Sodium Hypochlorite and Glutaraldehyde

**Figure 2:**
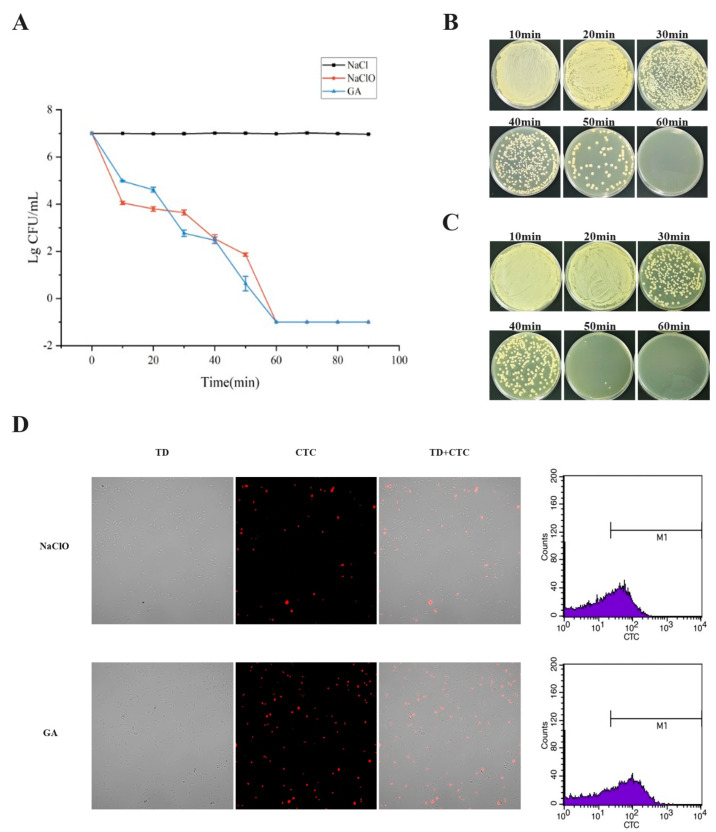
Induction of the viable but non-culturable state in *Klebsiella pneumoniae* by sodium hypochlorite or glutaraldehyde. (**A**) Dynamic changes in culturable cell numbers during treatment with 30 mg/L NaClO and 60 mg/L GA, with physiological saline-suspended cells as the control group. (**B**) Dynamic changes in culturable cell numbers during 30 mg/L NaClO treatment. (**C**) Dynamic changes in culturable cell numbers during 60 mg/L GA treatment. (**D**) Fluorescence confocal microscopy images and flow cytometry histograms of CTC-stained bacteria following 60 min treatment with 30 mg/L NaClO or 60 mg/L GA. Red fluorescence denotes CTC-positive cells with respiratory activity; the M1 gate represents the CTC-positive population.

Under 30 mg/L sodium hypochlorite treatment, culturability of *Klebsiella pneumoniae* declined progressively with exposure time (Figure 2A). Plate counting revealed abundant culturable colonies at 50 min; extension to 60 min resulted in complete disappearance of culturable colonies on LB plates (colony count < 1 CFU/mL), demonstrating entry into the non-culturable phase (Figure 2B). To assess whether these non-culturable cells retained physiological activity, CTC staining was performed. Confocal laser scanning microscopy showed numerous cells emitting red fluorescence signals, indicating that a substantial population retained respiratory metabolic activity and had not undergone complete death (Figure 2D). Quantitative flow cytometric analysis further demonstrated that in samples induced with NaClO for 1 h, the proportion of CTC-positive cells was 50.80% with a mean fluorescence intensity (MFI) of 59.17 (Figure 2D). These results collectively indicate that although *Klebsiella pneumoniae* became non-culturable on plates after 60 min of sodium hypochlorite exposure, a large fraction of cells preserved respiratory metabolic activity, fulfilling the defining criteria of the VBNC state.

Under 60 mg/L glutaraldehyde treatment, *Klebsiella pneumoniae* displayed a similar time-dependent loss of culturability. Culturable colonies were still detectable at 50 min, but none were recovered after 60 min (colony count < 1 CFU/mL), confirming entry into the non-culturable phase (Figure 2A,B). Confocal microscopy after CTC staining revealed numerous CTC-positive cells at this stage (Figure 2D). Flow cytometry confirmed that in GA-induced 1 h samples, the proportion of CTC-positive cells reached 63.44% with an MFI of 106.62—higher than in the NaClO group—indicating that a greater proportion of cells maintained respiratory activity despite loss of culturability (Figure 2D).

In summary, exposure to 30 mg/L NaClO or 60 mg/L GA for 1 h rendered *Klebsiella pneumoniae* non-culturable on plates (<1 CFU/mL) while preserving a high proportion of CTC-positive cells, supporting induction into the VBNC state. These conditions represent sublethal dosing and treatment durations comparable to or exceeding those encountered in real-world disinfection protocols, thereby conferring practical relevance [18,19]. All subsequent experiments employed these induction parameters, with plate spreading performed after each sample preparation to verify VBNC entry and exclude culturable-cell contamination.

### 3.3. Scanning Electron Microscopy of VBNC-State Klebsiella pneumoniae Induced by Sodium Hypochlorite and Glutaraldehyde

**Figure 3:**
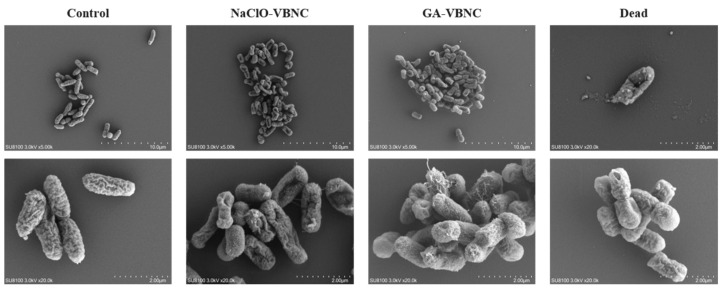
Scanning electron micrographs of untreated control *Klebsiella pneumoniae* (Control), VBNC-state cells induced by sodium hypochlorite or glutaraldehyde, and dead cells.

Scanning electron microscopy (SEM) revealed that untreated control (Control) *Klebsiella pneumoniae* displayed a typical short-rod morphology with clear, uniform cell outlines, relatively smooth and continuous surfaces, and no obvious collapse or rupture. Cells were dispersed without abnormal attachment structures or surface fibrillar protrusions (Figure 3).

Following 1 h exposure to 30 mg/L sodium hypochlorite (NaClO-VBNC), cells retained the basic rod shape without overt breakage, yet numerous cells exhibited pronounced surface depressions and irregular wrinkling. Some cells showed marked collapse-like deformation, increased surface roughness, and irregular edge contours. Intact bacterial bodies remained visible at the population level, although overall structures were distinctly contracted relative to the control. Bacteria killed by sodium hypochlorite exhibited severe structural damage accompanied by leakage of intracellular contents, followed by complete death and eventual fragmentation and lysis.

After 1 h treatment with 60 mg/L glutaraldehyde (GA-VBNC), cells likewise preserved the rod morphology, but surface topography underwent more intricate alterations. In addition to mild collapse in some cells, prominent whisker-like or fibrillar structures appeared on several bacterial surfaces, accompanied by net-like attachments in localized areas. Surface granularity increased markedly, rendering structures obviously rough. Compared with the sodium hypochlorite group, glutaraldehyde-treated cells displayed more pronounced “fixed” morphology, with some cells appearing relatively turgid yet bearing evident surface deposits. In contrast, bacteria killed by glutaraldehyde also showed membrane damage and loss of intracellular contents once their tolerance limit was exceeded. However, because glutaraldehyde exerts a cross-linking and fixation effect on cellular structures, the outer cell envelope remained relatively full and intact in appearance, without the pronounced collapse or lytic disruption observed in the sodium hypochlorite-treated cells.

Neither disinfectant induced widespread cell rupture or lysis; intact bacterial structures were retained, consistent with entry into the VBNC state without complete death. Nevertheless, the surface micro-morphology of VBNC-state *Klebsiella pneumoniae* underwent substantial remodeling, and clear morphological distinctions existed between sodium hypochlorite- and glutaraldehyde-induced VBNC populations, suggesting that the underlying induction mechanisms differ.

### 3.4. Effects of Different Additives on Resuscitation of VBNC Klebsiella pneumoniae and Associated Growth Kinetics

To assess the impact of various resuscitation conditions on growth recovery of VBNC-state *Klebsiella pneumoniae*, OD600 was recorded every 30 min using a temperature-controlled automatic microplate reader over a 25 h period, enabling comparison of growth kinetic profiles across treatments. Negative controls maintained consistently low OD backgrounds without sustained increases, while positive controls displayed classic proliferation curves, confirming negligible contamination and reliable monitoring. Overall, the promotional efficacy of different additives differed markedly between NACLO-VBNC (sodium hypochlorite-induced) and GA-VBNC (glutaraldehyde-induced) populations: NaClO-VBNC exhibited pronounced OD rises and plateau formation under several additives, whereas GA-VBNC responses were more restricted to specific substrates, indicating distinct resuscitation-limiting factors for the two VBNC types. These differential growth trends under identical additives may further reveal whether resuscitation proceeds via shared metabolic regulatory pathways.

**Figure 4:**
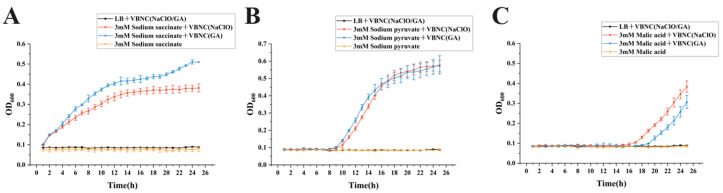
Resuscitation Effect Curves of Energy Metabolism-Related Additives. (**A**) 5 mM sodium succinate; (**B**) 3 mM sodium pyruvate; (**C**) 3 mM malic acid.

Within the energy metabolism reinforcement pathway, sodium succinate, sodium pyruvate, and malic acid all triggered resuscitation of VBNC cells (Figure 4A–C). Among them, sodium succinate produced the strongest effect: across repeated experiments, OD600 began rising continuously within approximately 2 h, followed by stable growth and plateau formation, yielding the shortest lag phase and rapid resuscitation initiation. In contrast, sodium pyruvate and malic acid groups typically required an 8–12 h stagnation period before a detectable OD600 increase and subsequent proliferation.

**Figure 5:**
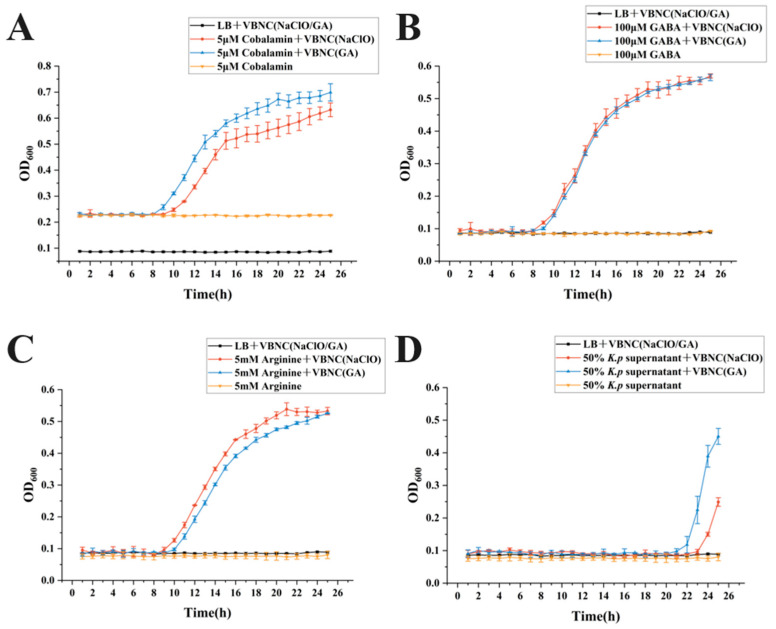
Resuscitation Effect Curves of Additives Related to Cell Structure Stabilization and Signal Regulation. (**A**) 5 μM cobalamin; (**B**) 100 μM GABA; (**C**) 5 mM arginine; (**D**) 50% *K. pneumoniae* cell-free supernatant.

At the quorum-sensing and growth-signaling level, GABA, L-arginine, and cobalamin (vitamin B12) each promoted VBNC resuscitation to varying degrees, evidenced by repeatable OD600 increases and plateau formation within the monitoring window (Figure 5). Cell-free supernatant from *Escherichia coli MG1655*, a high AI-2 producer, served as an exogenous quorum-sensing signal source [20,21]; however, no resuscitation of VBNC *Klebsiella pneumoniae* was observed across addition ratios (Figure 5D). In contrast, cell-free supernatant from *Klebsiella pneumoniae* itself elicited the most distinctive promotional effect, with stable and repeatable resuscitation growth observed at supernatant addition ratios of 30–50%.

**Figure 6:**
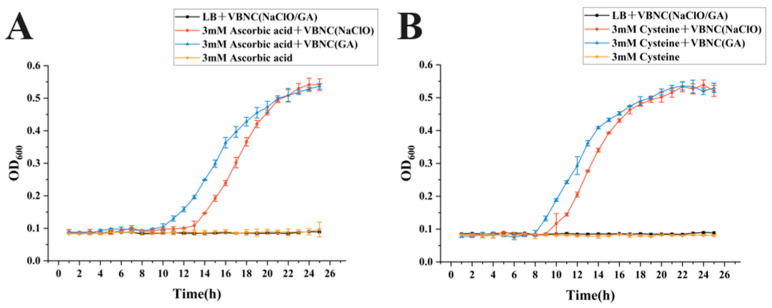
Resuscitation Effect Curves of Antioxidant and Repair-Related Additives. (**A**) 3 mM ascorbic acid; (**B**) 3 mM cysteine.

Beyond energy metabolism and quorum-sensing/growth signaling pathways, additives supporting antioxidant defense and metabolic repair also provided auxiliary resuscitation benefits. 3 mM L-cysteine and 3 mM ascorbic acid (vitamin C) induced stable resuscitation in both VBNC types, with kinetic profiles comparable to sodium pyruvate (Figure 6). However, at the cellular structure and functional stability level, magnesium chloride (MgCl_2_) failed to elicit resuscitation, and additional additives warrant evaluation in subsequent studies.

Among the additive groups confirmed to induce resuscitation in the present study, sodium succinate demonstrated the optimal overall performance with respect to initiation time, resuscitation intensity, and repeatability/stability. It is therefore identified as the most efficient, rapid, and stable resuscitation additive in this system and was subsequently employed as the resuscitation sample for all further experimental investigations.

### 3.5. Metabolic Activity of Klebsiella pneumoniae in the VBNC State and After Resuscitation

To evaluate the impact of sodium hypochlorite- and glutaraldehyde-induced VBNC states and sodium succinate resuscitation on the metabolic function of *Klebsiella pneumoniae*, intracellular reactive oxygen species levels and ATP content were quantified in each group. The viable cell control and dead cell control served as references for normal metabolic activity and complete inactivation, respectively; the sodium hypochlorite-induced VBNC group (NaClO-VBNC) and glutaraldehyde-induced VBNC group (GA-VBNC) reflected metabolic features in the non-culturable state; the sodium hypochlorite + sodium succinate resuscitation group (RCL) and glutaraldehyde + sodium succinate resuscitation group (RGA) reflected the extent of metabolic recovery after resuscitation.

#### 3.5.1. Intracellular Reactive Oxygen Species Levels

**Figure 7:**
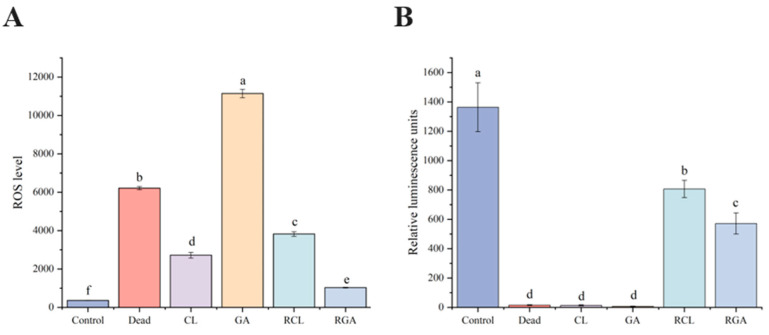
Reactive oxygen species levels and ATP content. (**A**) Intracellular reactive oxygen species levels (relative fluorescence intensity). (**B**) Intracellular ATP content (relative luminescence units). Control, untreated viable cells; Dead, heat-killed control; CL, sodium hypochlorite-induced VBNC cells; GA, glutaraldehyde-induced VBNC cells; RCL, CL resuscitation group; RGA, GA resuscita-tion group. Different letters indicate statistically significant differences between groups; identical letters indicate no significant difference (one-way ANOVA with Tukey’s multiple comparisons test, *p* < 0.05).

Intracellular ROS levels were measured using DCFH-DA fluorescence intensity, as shown in Figure 7A. The viable-cell control exhibited the lowest ROS fluorescence intensity (360.50 ± 8.55), indicating a low level of oxidative stress under normal physiological conditions. In contrast, the dead-cell control showed a sharp increase to 6209.83 ± 91.17, representing a 16.2-fold increase relative to the viable-cell control (*p* < 0.001). The mean ROS level in the NaClO-VBNC group was 2720.67 ± 152.02, which was significantly higher than that in the viable-cell control (*p* < 0.001) but significantly lower than that in the dead-cell control (*p* < 0.001). The mean ROS level in the GA-VBNC group reached 11,142.50 ± 228.34, which was significantly higher than those in both the viable-cell control and dead-cell control (both *p* < 0.001).

After resuscitation, the ROS level in the RGA group decreased sharply to 1030.50 ± 20.73, which was significantly lower than that in the GA-VBNC group (*p* < 0.001) and approached the level of the viable-cell control. In contrast, the ROS level in the RCL group increased to 3823.50 ± 122.39 after resuscitation, which was significantly higher than that in the NaClO-VBNC group (*p* < 0.001) but still lower than that in the dead-cell control (*p* < 0.001). These opposite ROS trends after resuscitation suggest that the two disinfectant-induced VBNC populations differed in their oxidative-stress responses during recovery.

#### 3.5.2. Intracellular ATP Levels

Bioluminescence assay was used to quantify relative intracellular ATP content (relative luminescence units, RLU), as shown in Figure 7B. The viable cell control exhibited the highest ATP level (mean 1263.67 ± 65.36), reflecting active energy metabolism under normal conditions. The dead cell control showed a drastic drop to near-background levels (mean 15.17 ± 2.48), representing a 98.8% reduction relative to the viable control, attributable to rapid ATP hydrolysis and content leakage after cell death.

Mean ATP in the NaClO-VBNC group was 12.83 ± 1.47 and in the GA-VBNC group 5.17 ± 1.17; both were significantly lower than the viable control (*p* < 0.001), indicating quiescent energy metabolism in VBNC cells induced by either disinfectant, approaching levels observed in dead cells.

Following sodium succinate resuscitation, ATP levels recovered markedly in both groups. The RCL group reached 757.83 ± 32.75 (60.0% of viable control, ~59-fold increase vs. NaClO-VBNC, *p* < 0.001). The RGA group reached 454.17 ± 57.10 (35.9% of viable control, ~88-fold increase vs. GA-VBNC, *p* < 0.001). Both groups thus achieved effective reconstruction of energy metabolism. Although absolute ATP recovery was higher in RCL, note that initial ATP levels were significantly higher in NaClO-VBNC than in GA-VBNC. Moreover, ATP levels in RCL remained significantly higher than in RGA (*p* < 0.001), indicating more complete restoration of ATP synthesis capacity in sodium hypochlorite-induced VBNC cells under sodium succinate treatment.

### 3.6. Antioxidant Enzyme Activities of Klebsiella pneumoniae in the VBNC State and After Resuscitation Induced by Sodium Hypochlorite and Glutaraldehyde

To further assess the impact of sodium hypochlorite- and glutaraldehyde-induced VBNC states and sodium succinate resuscitation on the bacterial antioxidant system, catalase (CAT) and superoxide dismutase (SOD) activities were measured in each group. The untreated viable cells served as Control, heat-killed cells as Dead, and the experimental groups comprised CL, GA, RCL, and RGA.

**Figure 8:**
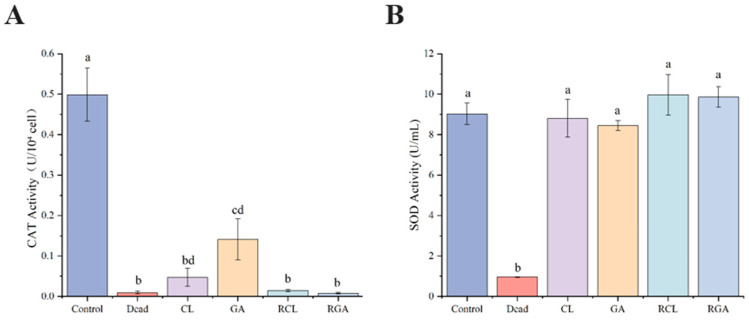
Antioxidant enzyme activities of *Klebsiella pneumoniae* in different physiological states. (**A**) Cat-alase activity. (**B**) Superoxide dismutase activity. Control, untreated viable cells; Dead, heat-killed control; CL, sodium hypochlorite-induced VBNC cells; GA, glutaraldehyde-induced VBNC cells; RCL, sodium hypochlorite-induced VBNC cells resuscitated with sodium succinate; RGA, glutaraldehyde-induced VBNC cells resuscitated with sodium succinate. Groups with the same letter are not significantly different, while groups with different letters are significantly different (*p* < 0.05).

CAT activity is expressed in U/10^4^ cells (Figure 8A). Control CAT was 0.499 ± 0.065. Compared with Control, CL CAT decreased markedly to 0.048 ± 0.022 (~9.5% of control). GA CAT also declined to 0.141 ± 0.051 (~28.3% of control), higher than CL yet still significantly lower than Control. Notably, CAT showed no recovery trend after resuscitation: RCL CAT fell further to 0.0148 ± 0.0028 and RGA to 0.0080 ± 0.0023, both approaching the Dead group level of 0.0094 ± 0.0035. Thus, CAT was already severely impaired or strongly suppressed during VBNC induction and remained low under resuscitation conditions, suggesting persistent inhibition or insufficient repair of the CAT pathway.

SOD activity is expressed in U/mL (Figure 8B). Control SOD was 9.03 ± 0.53; CL SOD was 8.81 ± 0.93 (close to control overall); GA SOD was 8.44 ± 0.25 (slight decline). After resuscitation, SOD activity increased: RCL reached 9.97 ± 1.01 and RGA 9.86 ± 0.50, both exceeding the control mean. Dead SOD was 0.960 ± 0.016, markedly lower than all other groups. SOD therefore remained generally near control levels during VBNC induction and showed upregulation after resuscitation, whereas CAT decreased sharply after induction and stayed near dead-cell reference levels after resuscitation.

### 3.7. Intracellular Protein Levels of Klebsiella pneumoniae in the VBNC State and After Resuscitation Induced by Sodium Hypochlorite and Glutaraldehyde

#### 3.7.1. BCA Quantification of *Klebsiella pneumoniae* Cellular Protein

**Figure 9:**
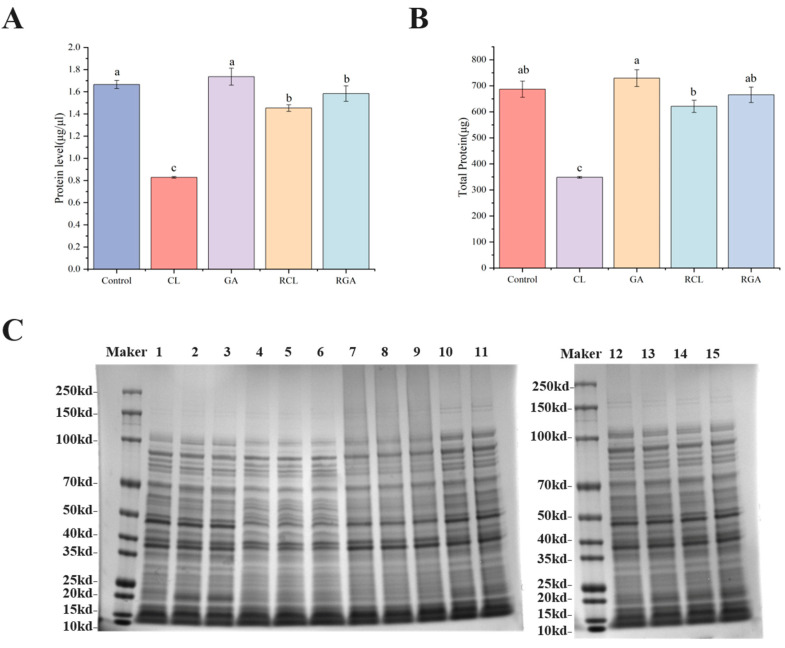
Total protein SDS-PAGE profiles and BCA quantification analysis of *Klebsiella pneumoniae* in different states. (**A**) Total protein concentration (μg/μL). (**B**) Total protein content (μg). (**C**) SDS-PAGE of total protein. Different letters indicate statistically significant differences between groups; identical letters indicate no significant difference (one-way ANOVA with Tukey’s multiple comparisons test, *p* < 0.05).

BCA quantification of *Klebsiella pneumoniae* cellular protein is shown in Figure 9. Control total protein concentration averaged 1.665 μg/μL and total protein amount 686.67 μg, reflecting normal physiological protein expression. In the CL group, total protein concentration averaged 0.828 μg/μL and total amount 348.33 μg, representing 50.3% and 49.3% reductions relative to Control, respectively, indicating that the sodium hypochlorite-induced VBNC state is accompanied by substantial loss of total protein content—consistent with oxidative damage-induced protein degradation, translational repression, and content leakage due to cell contraction or membrane injury. In the GA group, total protein concentration averaged 1.736 μg/μL and total amount 729.00 μg, close to and slightly higher than Control, suggesting that glutaraldehyde-induced VBNC cells did not undergo overt protein loss, possibly owing to the protein-stabilizing effect of glutaraldehyde cross-linking. After resuscitation, RCL total protein concentration reached 1.453 μg/μL and total amount 621.00 μg (75.4% and 78.3% increases vs. CL, recovering to 87.2% of Control concentration and 90.4% of total amount), demonstrating that sodium succinate resuscitation can promote protein re-expression and functional recovery to a certain extent. RGA total protein concentration was 1.583 μg/μL and total amount 665.33 μg (8.8% and 8.7% decreases vs. GA, yet overall close to Control), indicating that glutaraldehyde-induced VBNC cells did not experience significant additional protein synthesis bursts during resuscitation and that certain key functional proteins were preserved in the VBNC state.

#### 3.7.2. SDS-PAGE Electrophoresis Results

SDS-PAGE results are shown in Figure 9C. Loading volumes were normalized to equal total protein according to BCA quantification to accurately reflect relative abundance changes in specific proteins across samples. Lanes 1–3 (Control) displayed clear, complete protein band profiles spanning 10 kDa to >250 kDa with uniform intensity, reflecting the normal protein expression spectrum of *Klebsiella pneumoniae*. Lanes 4–6 (CL) showed markedly reduced overall band intensity compared with Control, especially in the high-molecular-weight region (>70 kDa) and medium-molecular-weight region (25–40 kDa), where bands were significantly fainter or absent; the low-molecular-weight region (<15 kDa) exhibited enhanced diffusion, indicating increased protein degradation products. This pattern closely matches the BCA quantification results and demonstrates that sodium hypochlorite-induced oxidative stress caused widespread protein damage and degradation. Lanes 7–9 (GA) showed an overall band profile highly similar to Control, with clear, complete bands, no obvious diffusion or loss, and good retention in the high-molecular-weight region. Individual bands in the 35–50 kDa region and near 25 kDa displayed slight intensity increases or minor shifts, reflecting conformational changes or partial upregulation caused by glutaraldehyde cross-linking. Overall, protein profile integrity in the GA group far exceeded that in the CL group, consistent with BCA results. Lanes 10–12 (RCL) showed significant band recovery compared with CL, with most weakened bands reappearing and the overall profile approaching Control. However, high-molecular-weight bands (100 kDa, 70 kDa) remained incompletely restored (intensity still slightly lower than Control), and low-molecular-weight diffusion was reduced relative to CL yet still slightly higher than Control, suggesting incomplete reconstruction of protein homeostasis. Lanes 13–15 (RGA) displayed a profile essentially identical to both Control and GA, with clear, complete bands and no overt abnormalities. Compared with GA, individual band intensities showed minor adjustments, but the overall profile remained stable, indicating smooth recovery of the protein expression spectrum in glutaraldehyde-induced VBNC cells after resuscitation without dramatic protein remodeling.

### 3.8. Cell Membrane Integrity and Permeability of Klebsiella pneumoniae in the VBNC State and After Resuscitation

**Figure 10:**
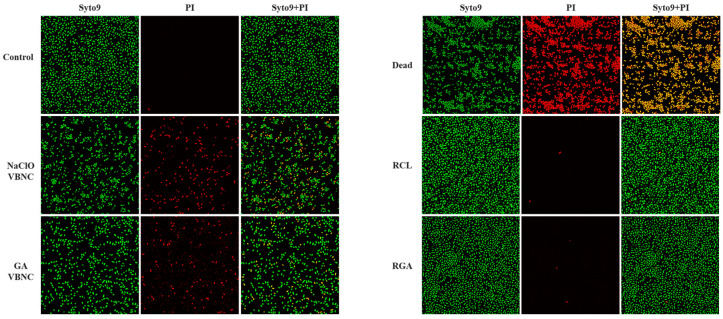
Fluorescence images of *Klebsiella pneumoniae* stained with SYTO9 and PI under different states.

To evaluate changes in cell membrane integrity and permeability of *Klebsiella pneumoniae* in the VBNC state and after resuscitation, SYTO9/PI double staining was performed and observed by fluorescence confocal microscopy (Figure 10). Control cells were predominantly stained green by SYTO9 with virtually no PI-positive signals, indicating intact membranes and low permeability. Compared with the control, both VBNC samples showed clear increases in PI-positive cells, confirming that a portion of cells indeed sustained irreversible membrane damage and death during induction. Nevertheless, within the same field, numerous cells exhibited only SYTO9 positivity, demonstrating that despite loss of culturability, a substantial proportion of the VBNC population retained membrane integrity without rupture. This further confirms that after loss of culturability, the majority of cells did not die but entered the VBNC state.

NaClO-induced VBNC samples displayed more pronounced PI-positive signals, indicating stronger disruption of membrane permeability; conversely, GA-induced VBNC samples showed a relatively higher proportion of green fluorescent cells, indicating better preservation of membrane barrier function. These observations align with the aforementioned flow cytometry results and further demonstrate that the two disinfectants induce VBNC via distinct primary damage pathways—NaClO predominantly triggers oxidative membrane injury, while GA inhibits key physiological processes through cross-linking, allowing cells to enter a non-culturable state with relatively preserved membrane structure. Compared with the control, VBNC cells overall appeared slightly shortened with reduced volume, consistent with stress-induced contraction and decreased membrane fluidity [22].

In the resuscitation samples, both RCL and RGA groups were dominated by SYTO9-positive cells, with markedly reduced PI-positive signals and cell density/morphology distributions approaching those of the control, indicating restoration of membrane barrier function after resuscitation. Combined with the aforementioned resuscitation results, SYTO9/PI confocal observation supports that VBNC cells can return to a state of near-normal membrane integrity and permeability under appropriate conditions (Table 2).

**Table 2:** Summary Table of Experimental Results.

Comparison Dimension	Parameter	Sodium Hypochlorite (NaClO) Group	Glutaraldehyde (GA) Group
Induction Conditions	Concentration and exposure time	30 mg/L, 60 min	60 mg/L, 60 min
Culture Characteristics	Plate count (CFU/mL)	Non-culturable	Non-culturable
Cell Viability	CTC-positive rate (flow cytometry)	50.80%	63.44%
Micromorphology	SEM observations	Surface collapse and shrinkage (oxidative damage)	Filamentous and network-like structures (protein cross-linking)
Energy Metabolism	ATP level in the VBNC state	Extremely low (close to the dead-cell control)	Extremely low (close to the dead-cell control)
	ATP recovery rate after resuscitation	60.0% (relatively high recovery)	35.9%
Oxidative Stress	ROS level in the VBNC state	Moderately high	Extremely high
	ROS trend after resuscitation	Continues to rise	Rapidly returns to normal
Antioxidant Enzymes	CAT activity	Severely impaired (only 9.5%)	Partially impaired (28.3%)
	SOD activity	Remains stable, with compensatory upregulation after resuscitation	Remains stable, with coordinated recovery after resuscitation
Proteomic Profile	Total protein content	Reduced by 50%; significant degradation/loss	Remains stable; close to that of normal bacteria
	SDS-PAGE bands	High-molecular-weight bands disappeared; low-molecular-weight bands became diffuse	Bands remained intact and highly similar to those of normal bacteria
Resuscitation Characteristics	Optimal additive	Sodium succinate	Sodium succinate
	Repair pattern	Unbalanced repair (persistent ROS imbalance)	Homeostatic repair (all indicators return to normal synchronously)

## 4. Discussion

The present study systematically compared the differences in VBNC induction by sublethal concentrations of sodium hypochlorite (NaClO) and glutaraldehyde (GA) using *Klebsiella pneumoniae *FY170-1 as the model organism, establishing a comprehensive evidence chain across culturability, membrane integrity, respiratory activity, ultrastructure, resuscitation kinetics, energy metabolism, antioxidant enzyme activity, and protein expression. Treatment with 30 mg/L NaClO or 60 mg/L GA for 60 min completely eliminated culturability; however, CTC staining demonstrated that 50.80% and 63.44% of cells, respectively, retained respiratory metabolic activity, and SYTO 9/PI staining confirmed preservation of membrane integrity in the majority of cells, fulfilling the classical definition of the VBNC state. Nevertheless, the VBNC populations induced by the two disinfectants displayed systematic differences in morphology, metabolism, antioxidant capacity, and protein homeostasis. These distinctions not only highlight divergent mechanisms of action but also provide critical insights into the factors limiting VBNC resuscitation.

Scanning electron microscopy first rendered these differences tangible. NaClO-treated cells retained an overall rod morphology but exhibited widespread surface depressions and irregular wrinkling with collapse-like deformation, consistent with cytoplasmic contraction and reduced membrane tension caused by intense oxidation; importantly, no overt rupture occurred, aligning with membrane integrity staining results. In contrast, GA-treated cells developed prominent filamentous and net-like surface structures accompanied by increased roughness and granularity. Given GA’s protein-cross-linking properties, these features most likely represent fimbriae and capsular polysaccharides fixed in place and visualized under electron microscopy, indicating that while GA inhibits division and certain functions, it stabilizes surface architecture. Neither VBNC population underwent widespread lysis, thereby providing the structural foundation for subsequent resuscitation.

Resuscitation kinetic experiments further clarified differences in the ease of metabolic reactivation. Among the tested additives, sodium succinate demonstrated the fastest and most consistent promotional effect, initiating sustained OD600 increases within ~2 h, whereas other energy substrates such as sodium pyruvate and malic acid typically required an 8–12 h lag phase before growth commenced. Prior studies have shown that pyruvate salts promote VBNC resuscitation in multiple bacteria; sodium pyruvate likewise produced stable resuscitation in the present work, an effect attributable to hydrogen peroxide scavenging, oxidative stress relief, and initiation of biosynthesis as a usable carbon source [23,24,25]. This contrast underscores that the primary bottleneck for resuscitation is energy metabolism restart: as a tricarboxylic acid cycle intermediate, sodium succinate directly feeds the electron transport chain to drive ATP synthesis, bypassing potentially compromised upstream steps. Contrary to reports proposing AI-2 as a VBNC resuscitation promoter [20,26], cell-free supernatant from the high-AI-2-producing *Escherichia coli MG1655* failed to elicit resuscitation of VBNC *K. pneumoniae* across addition ratios, indicating that the heterologous system was inadequate under the conditions tested. Strikingly, cell-free supernatant from *K. pneumoniae* itself produced the most distinctive resuscitation response: at 30–50% addition ratios, stable and reproducible growth was observed. Its kinetics differed markedly from energy substrates, featuring the longest lag phase yet the highest growth rate once resuscitation began. Published data indicate that homologous cell-free supernatants can resuscitate VBNC bacteria; the present results confirm this for *K. pneumoniae* and reveal a unique kinetic profile, suggesting activation of metabolism via multiple synergistic resuscitation-promoting factors [27,28]. Taken together, these findings further imply that homologous *K. pneumoniae* supernatant may contain species-specific signaling molecules beyond AI-2, or intraspecific quorum-sensing signals that are particularly effective in triggering VBNC recovery, thereby providing an important direction for future studies on the molecular basis of resuscitation. Notably, although NaClO-induced VBNC cells ultimately restored growth under sodium succinate, initiation was consistently later than in the GA group and responses to certain additives were weak in the CL group, implying deeper metabolic injury or a higher repair threshold in NaClO-VBNC cells.

Quantitative ATP and ROS data provided direct biochemical corroboration. ATP levels in both VBNC populations plummeted to near-dead-cell values, reflecting profound energy-flux compression in the VBNC state. Post-resuscitation, ATP recovered significantly in both groups, confirming effective metabolic restart by sodium succinate; however, absolute recovery and fold increases differed: RCL reached 60.0% of control (59-fold rise versus NaClO-VBNC), while RGA reached 35.9% (88-fold rise versus GA-VBNC). Combined with faster initiation in RGA, these patterns suggest that GA-induced damage to energy-metabolism enzymes is predominantly reversible inhibition that rapidly reverses upon substrate availability, whereas NaClO-induced oxidative damage involves irreversible modifications requiring additional repair steps. ROS dynamics offered an even sharper contrast: NaClO-VBNC ROS rose moderately to 7.5-fold viable-cell levels and further increased to 10.6-fold after resuscitation, whereas GA-VBNC ROS surged to 30-fold but plummeted to near-normal levels post-resuscitation. This apparent paradox precisely delineates the two damage types. NaClO’s potent oxidation likely inflicts irreversible injury on the respiratory chain and antioxidant enzymes; although sodium succinate reactivates the chain, unrepaired oxidative stress generates excess oxidants and the antioxidant system fails to keep pace, resulting in sustained ROS accumulation. GA primarily inhibits enzymes via cross-linking with minimal direct protein destruction; upon resuscitation, cross-links are relieved or metabolic flow resumes, enabling rapid enzyme reconstruction and swift relief of oxidative pressure. Importantly, the supranormal ROS fluorescence in GA-VBNC likely stems partly from altered outer-membrane permeability caused by GA cross-linking, which enhances DCFH-DA uptake or retention rather than reflecting a genuinely higher ROS burden; therefore, interpretation must integrate complementary indicators [29,30,31].

Antioxidant enzyme assays further supported this mechanistic interpretation. CAT activity in NaClO-VBNC had already fallen to 9.5% of the control level and declined further in RCL to a level close to that of dead cells, highlighting the marked vulnerability of this heme-containing enzyme to oxidative damage [32,33,34]. This difference may be related to structural and functional distinctions between CAT and SOD. As a heme-containing enzyme, CAT is more susceptible to oxidative injury at its porphyrin iron center and surrounding residues, particularly under strong oxidant exposure [35]. By contrast, SOD contains a more tightly coordinated metal center and generally exhibits greater structural stability under oxidative stress, which may contribute to its relatively preserved activity. Consistent with this, SOD activity in NaClO-VBNC remained comparable to the control level but increased significantly above the control level after resuscitation in RCL, suggesting a compensatory response to persistent superoxide stress. However, preservation or upregulation of SOD alone is insufficient to ensure effective ROS detoxification, because the hydrogen peroxide generated downstream of SOD activity requires subsequent removal by CAT or other peroxide-scavenging systems [36]. Therefore, severe CAT impairment may have disrupted antioxidant balance and contributed to the paradoxical ROS increase observed in RCL [37,38]. In GA-VBNC, CAT activity decreased to 28.3% of the control level, remaining substantially higher than that in NaClO-VBNC, and recovered markedly in RGA together with restoration of SOD activity. These results suggest that GA-induced cells experienced relatively milder antioxidant-enzyme damage and showed a more coordinated recovery of redox-regulating capacity during resuscitation.

Protein quantification and SDS-PAGE electrophoresis provided molecular-level confirmation. Total protein in NaClO-VBNC declined ~50% relative to control; SDS-PAGE revealed marked weakening of high-molecular-weight bands and enhanced diffusion in the low-molecular-weight region, consistent with oxidative degradation. After resuscitation, RCL total protein recovered to >90% of control, yet high-molecular-weight bands remained incompletely restored and low-molecular-weight diffusion persisted, indicating incomplete protein homeostasis—mirroring sustained oxidative pressure. In contrast, GA-VBNC total protein was comparable to control with intact, clear bands and only minor shifts, reflecting primarily cross-linking modification rather than degradation; RGA profiles recovered smoothly without abnormalities, reinforcing the reversibility of GA-induced damage.

Collectively, the present study delineates a complete evidence chain from induction to resuscitation: NaClO drives a “metabolically damaged” VBNC state via oxidative injury that causes protein degradation, CAT inactivation, and irreversible respiratory-chain damage; although energy metabolism partially recovers after resuscitation, redox homeostasis cannot be synchronously restored, yielding a non-equilibrium repair pattern. GA induces a “metabolically frozen” VBNC state through cross-linking inhibition that preserves protein architecture with mainly reversible damage; after resuscitation, energy and antioxidant systems are coordinately restored, rapidly regaining homeostasis. The distinction between the two VBNC states is therefore mechanistic rather than merely quantitative, with profound implications for disinfection practice and food-safety risk assessment.

First, reliance on plate-culture negativity as the sole criterion for disinfection success may severely underestimate viable-cell risk. In this study, both disinfectants produced sterile plates after 60 min, yet CTC staining revealed that more than half the cells retained metabolic activity and could resuscitate in the presence of common environmental metabolites such as sodium succinate. Consequently, culture-negative results alone may conceal VBNC cells as hidden contamination sources capable of resuming proliferation and re-entering the food chain or infecting hosts once conditions become favorable. Second, VBNC cells induced by different disinfectants exhibit distinct resuscitation potentials and risk profiles. NaClO-induced VBNC maintains persistently elevated ROS after resuscitation, potentially elevating DNA damage and resistance mutation risks, whereas GA-induced VBNC resuscitates more completely and rapidly, with preserved surface structures that may enhance adhesion and persistence; thus, sublethal exposures should be minimized in practice, and disinfectant type and concentration should be tailored to the target. Third, ubiquitous environmental organic acids (e.g., succinate and pyruvate salts) can act as VBNC awakening signals; elevated organic-matter levels after disinfection will markedly increase resuscitation risk—a concern particularly relevant to food-processing environments and healthcare settings.

## 5. Conclusions

This study successfully established experimental models for NaClO- and GA-induced VBNC states in *K. pneumoniae* and elucidated mechanistic differences between the two disinfectants across multiple dimensions. NaClO-induced VBNC is characterized primarily by oxidative damage, manifested as severe CAT impairment, protein degradation, surface depressions, extremely low ATP, and moderately elevated ROS; after resuscitation, energy metabolism partially recovers but oxidative stress persists, CAT fails to rebuild, and SOD is compensatorily upregulated, resulting in a non-equilibrium repair pattern. GA-induced VBNC is characterized primarily by cross-linking inhibition, manifested as partial CAT impairment, stable total protein, fixed surface structures, lower ATP, rapid ROS normalization after resuscitation, and synchronous CAT/SOD recovery, yielding a steady-state repair pattern. Sodium succinate serves as a highly efficient resuscitation trigger by directly entering the tricarboxylic acid cycle to restart energy metabolism; however, the divergent resuscitation pathways highlight that oxidative damage is less reversible than cross-linking inhibition. These findings confirm that sublethal disinfectant exposure can induce *K. pneumoniae* into the VBNC state with subsequent resuscitation under favorable conditions, challenging current culture-based disinfection evaluation systems and advocating the adoption of multi-dimensional detection methods for accurate assessment of efficacy and risk. Future studies should further dissect the molecular regulatory networks governing VBNC formation and resuscitation and evaluate the pathogenic potential of resuscitated strains, thereby furnishing scientific evidence for food safety and hospital infection control.

## Figures and Tables

**Table 1:** Resuscitation additives based on different metabolic pathways.

Functional Levels of Resuscitation Additives	Additive	Concentration
Energy Metabolism Reinforcement Additives	Sodium succinate	1–5 mM
	Sodium pyruvate	0.1–10 mM
	Sodium L-malate	1–5 mM
	Yeast extract	0.3–0.5% (*w*/*v*)
	Glucose	0.2–0.5% (*w*/*v*)
	TSB broth	Prepared according to standard formulation
	BHI broth	Prepared according to standard formulation
Antioxidant and Repair Support Additives	Ascorbic acid (Vitamin C)	0.5–2 mM
	L-Cysteine	0.5–2 mM
Cellular Structure and Functional Stability Additives	Magnesium chloride (MgCl_2_)	1–3 mM
Quorum Sensing and Growth Signaling Additives	γ-Aminobutyric acid (GABA)	10–500 μM
	L-Arginine	0.5–5 mM
	Vitamin B12 (cobalamin)	1 nM–1 μM
	Autoinducer-2 (*E. coli MG1655* culture supernatant)	5–50% (*v*/*v*)
	Autologous culture supernatant (*K. pneumoniae* cell-free supernatant)	5–50% (*v*/*v*)

## Data Availability

The original contributions presented in this study are included in the article. Further inquiries can be directed to the corresponding authors.

## Abbreviations

The following abbreviations are used in this manuscript:

VBNC	viable but nonculturable
GA	Glutaraldehyde
RCL	Resuscitated NaClO-VBNC cells
RGA	Resuscitated GA-VBNC cells
NaClO-VBNC	NaClO-induced VBNC state
GA-VBNC	Glutaraldehyde-induced VBNC state
ROS	Reactive oxygen species
ATP	Adenosine Triphosphate
SOD	Superoxide Dismutase
CAT	Catalase
